# Unlocking the Use of LiCl as an Inexpensive Salt for Lithium-Ion Batteries with a Novel Anion Receptor

**DOI:** 10.3390/ma17133244

**Published:** 2024-07-02

**Authors:** Manabu Hirasawa, Akihiro Orita, Tsubasa Mimuro, Shin-ichi Kondo

**Affiliations:** 1 Institute for Advanced Integrated Technology, Resonac Corporation, 48 Wadai, Tsukuba 300-4247, Ibaraki, Japan; orita.akihiro.xiasy@resonac.com; 2 Department of Chemistry, Faculty of Science, Yamagata University, Yamagata 990-8560, Yamagata, Japan; s221702d@st.yamagata-u.ac.jp

**Keywords:** lithium-ion battery, anion receptor, lithium salt, lithium chloride, Li_4_Ti_5_O_12_/Li battery, electrolyte

## Abstract

Lithium chloride (LiCl) is an inexpensive and environmentally friendly salt abundant in the ocean. However, the insolubility of LiCl in conventional electrolyte solvents prevents the practical use of LiCl for lithium-ion batteries. Here, we report a novel method to increase the solubility of LiCl in a conventional electrolyte. The solubility of LiCl in ethylene carbonate (EC)/dimethyl carbonate (DMC) (1/1, *v*/*v*) is about quadrupled by adding a small amount of anion receptor with two urea moieties as recognition sites connecting with an ether chain. Anion receptor is an organic molecule that can associate with anions. Our anion receptor is able to associate with chloride anion. The ionic conductivity of LiCl in EC/DMC increased from 0.023 mS cm^−1^ (without an anion receptor) to 0.075 mS cm^−1^ (with a 0.05 M anion receptor). The electrolyte in the presence of a 0.05 M receptor exhibits higher ionic conductivity, rate capability, and cyclability than the electrolyte without the receptor.

## 1. Introduction

Lithium-ion batteries (LIBs) are devices that can convert and store electrical energy into chemical energy. LIBs are used in various applications, such as smartphones and electric vehicles, and have become indispensable to our lives. Much research has been conducted to improve battery properties such as energy density, cyclability, and rate capability. In particular, electrolytes affect the cyclability and the rate capability. In electrolytes, the Li-salts, as well as the organic solvent, have important roles in LIBs. Generally, LiPF_6_ is used as the common salt, while several alternative Li-salts have been reported, such as LiBF_4_ [1], lithium bis(trifluoromethanesulfonyl)imide (LiTFSI) [2], lithium bis(fluorosulfonyl)imide (LiFSI) [3], and LiClO_4_ [4]. However, most of the papers have mainly focused on enhancing the battery properties, not on the environmental friendliness or the cost. Even after LIBs have become very common, the demand for cost reduction remains strong. One of the reasons for the high cost of LIBs is ascribed to the high price of LiPF_6_, which is generally used as a salt in an electrolyte. LiPF_6_ is also known to be hydrolyzed by moisture, resulting in the formation of hydrofluoric acid, which can also negatively affect battery performance, such as gas formation within a battery and the deterioration of battery capacity [5]. The use of inexpensive and moisture-stable salts such as LiCl can be proposed to overcome the above problems. LiCl is easy to produce, economically friendly, chemically stable, and even exists in the ocean. However, LiCl has not been used for commercial LIB electrolytes due to its poor solubility in low-polarity solvents such as ethylene carbonate (EC) and dimethyl carbonate (DMC).

The organic molecule that can associate with anions is generally called an ‘anion receptor’ [6,7,8,9,10,11,12,13,14,15,16]. Recently, the addition of anion receptors to lithium-ion battery electrolytes has shown to increase the solubility of lithium salts and improve the ionic conductivity and transference numbers. In particular, boron-based anion receptors, which use Lewis acid as the recognition site for anions, have been widely studied to utilize lithium fluoride (LiF) or lithium oxides (Li_2_O and Li_2_O_2_), which are poorly soluble in electrolyte solvents such as DMC and propylene carbonate (PC) [17]. However, the effect of anion receptors using hydrogen bonds as recognition sites has been less explored [18,19].

**Figure 1:**
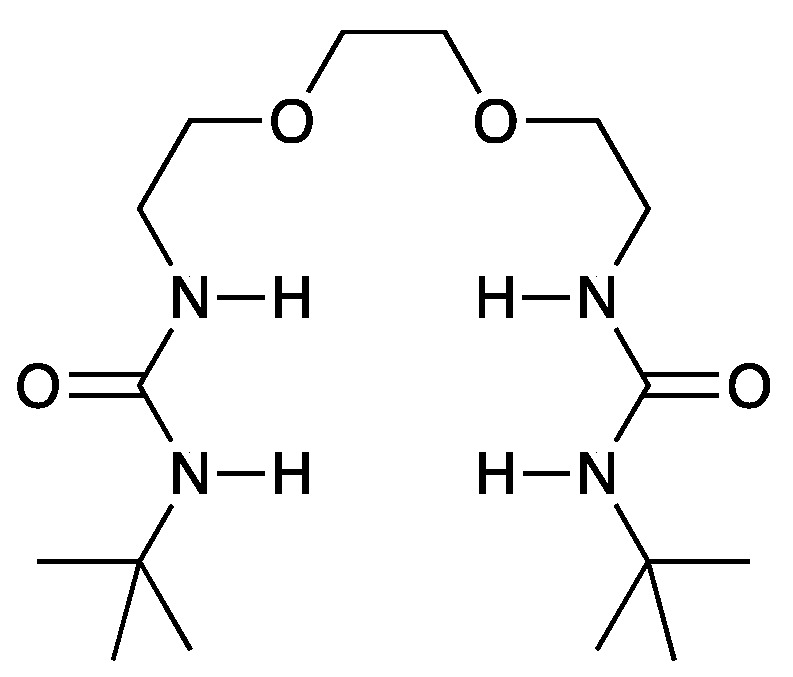
The structure of anion receptor (1,2-bis(2-(3-*tert*-butylureid)ethoxy)ethane).

We have reported novel anion receptors’ synthesis and recognition properties associated with hard anions such as F^−^ and Cl^−^ [12,13,14,15,16]. We have also reported flexible anion receptors with relatively high solubility and association ability, as illustrated in Figure 1 [20]. The receptor can associate with an anion such as Cl^−^ by multiple hydrogen bonds with NH groups of the urea moieties, forming the charge–delocalized complex. The complex is expected to have a weak Lewis-base, such as a soft anion like PF_6_^−^ with higher solubility in organic solvents. Therefore, the increase in solubility may increase the ionic conductivity of the electrolyte. If the ionic conductivity is sufficient to work LIBs, it can expand the possibilities of salts such as LiCl, which have not been generally used so far. This research reports the novel electrolyte based on a receptor with LiCl in EC and DMC as LIB electrolytes. EC and DMC have commercially used electrolyte solvents. We selected Li_4_Ti_5_O_12_ (LTO) as the electrode because a half-cell with a graphite electrode cannot work in our system, and LTO has been shown to work even with a highly concentrated LiCl/H_2_O electrolyte [21].

## 2. Materials and Methods

### 2.1. Fabrication of Materials

EC and DMC (Kishida Chemical, lithium battery grade) were used without purification. LiCl (FUJIFILM Wako Chemicals (Osaka, Japan), special grade) was used after being dried at 120 °C under vacuum for 12 h. The saturated LiCl electrolytes were prepared by mixing a given amount of LiCl. The urea-type anion receptor, 1,2-bis(2-(3-*tert*-butylureid)ethoxy)ethane, was synthesized according to the previously reported procedures [6]. A mixture of 1,2-bis(2-aminoethoxy)ethane (500 mg, 3.37 mmol) and *tert*-butyl isocyanate (735 mg, 2.2 eq) in dry tetrahydrofuran (6 mL) was refluxed under Ar atmosphere overnight. The mixture was cooled to 0 °C to give colorless solids as the product (804 mg, 69%). The LiCl and the receptor were mixed with solvents in an Ar-filled glove box. The prepared electrolytes are shown in Table 1. Although 0.5 M of LiCl was added to the EC/DMC (1/1 (*v*/*v*)), LiCl remained undissolved in the electrolytes, and the supernatant liquid was used after filtration. To verify the effect of anion receptor concentration, we added 0.1 M of the receptor to a solution containing the amount of 0.5 M LiCl. However, there was residue remaining undissolved. In addition, preparation in increments of 0.01 M is difficult due to preparation errors. Therefore, an anion receptor concentration of 0.05 M was chosen for this experiment.

The electrodes were fabricated by mixing the active materials of Li_4_Ti_5_O_12_ (LTO, UBE (Tokyo, Japan), SG), polyvinylidene difluoride (PVdF, Kureha battery materials (Tokyo, Japan), #9130), and acetylene black (AB, Denka (Tokyo, Japan), HS-100) in *N*-methylpyrrolidone in a weight ratio of 91:5:4 (LTO/PVdF/AB). The slurry was cast on an Al foil (20 μm thickness) for the LTO electrode using a doctor blade with a 150 μm gap. The coating amount was 100 g m^−2^. The electrodes were dried at 120 °C under vacuum for 12 h. The density of the electrodes was 1.9 g cm^−3^ after pressing and calendaring.

The LTO electrodes were punched to 16 mm φ. The Li foil was punched to 15 mm φ from a Li sheet roll (Honjo Chemical (Osaka, Japan), thickness: 0.5 mm, width: 48 mm). The electrodes and foils were used for the 2032 type coin cells. The injected amount of the electrolytes was 50 μL. The assembly and injection were performed in a dry room with a dew point below −30 °C. The cell structure is indicated in Figure S1. The counter electrode was Li.

### 2.2. Electrolyte Characterization

The ionic conductivity measurement was performed with the four-electrode cells (EC FRONTIER (Kyoto, Japan), S140, Pt|electrolyte|Pt) (Figure S2). The assembly was conducted in a glove box under an Ar atmosphere with a dew point of −40 °C or below. The alternating current (AC) impedance measurement of a cell was conducted using a Potentio/Galvanostat VSP (Bio-Logic (Seyssinet, France)). The measurement frequency was 1 MHz to 10 mHz, and the amplitude voltage was 10 mV. The ionic conductivity was calculated using Equation (1). A 0.01 M KCl aqueous solution (1413 µS cm^−1^) was used to calculate the cell constant, which was found to be C = 0.79 cm^−1^.

(1) σ=C/R

σ: ionic conductivity (S cm^−1^), R: resistance value (read value) (Ω), C: cell constant (cm^−1^).

The Li concentration in a saturated LiCl solution was determined with an inductively coupled plasma optical emission spectrometer (ICP-OES, Agilent 5100 manufactured by Agilent Technologies (Santa Clara, CA, USA)). After adding an excess amount of nitric acid to 0.3 g of the organic electrolyte sample, the sample was heated to 200 °C using a microwave sample pre-treatment device (ETHOS EASY manufactured by Milestone (Sorisole, Italy)). After cooling to room temperature, the sample was diluted to 30 g with ultrapure water and used as the measurement sample. A constant amount of the above sample was intermittently injected into the ICP-OES. The Li concentration (mol L^−1^) was calculated from the obtained concentration data (ppm) using Equation (2).

(2) $$C_{Li}/M_{Li}\times 100={C^{\prime }}_{Li}$$

$C_{Li}$: Li concentration (ppm (=mg L^−1^)), $M_{Li}$: molecular weight of Li = 6.94 (g mol^−1^), ${C^{\prime }}_{Li}$: Li concentration (mol L^−1^).

### 2.3. Electrochemical Measurements

The initial characteristics of the batteries were evaluated at 25 °C with a charge/discharge tester (Meiden Hokuto (Tokyo, Japan), HJ1005SD8). The cut-off voltages for charge and discharge were 1.0 V and 2.0 V, respectively. The charging protocol was conducted at a rate of 0.1 C constant current-constant voltage (CC-CV). The cut-off current was 1/50 C. The discharge protocol was conducted at a rate of 0.1 C constant current (CC). The protocols were performed for three cycles for initial testing.

The rate property was evaluated at 25 °C after initial tests. The charge rate test was conducted at a rate of 0.1−5 C CC. After each charge test, the cells were discharged at 0.1 C to set the state of charge to 0%. Discharge rate tests were conducted at 0.1−5 C CC. Before each discharge test, the cells were fully charged at 0.1 C CC-CV. The cut-off current was 1/50 C. The cycling test was done at the same charge/discharge setting as the initial tests.

## 3. Results

### 3.1. Ionic Conductivity Measurements

The ionic conductivity measurement was carried out to confirm that the addition of the anion receptor enhances the dissociation of LiCl. The results are shown in Table 2 and Figure S3. Although the ionic conductivity of electrolyte 2 without receptor was 0.023 mS cm^−1^ at room temperature, electrolyte 1 with receptor showed a value of 0.075 mS cm^−1^, which is 3.3 times higher than electrolyte 2, clearly indicating that the ionic conductivity of the saturated LiCl carbonate electrolyte increased with the addition of the anion receptor. The ionic conductivity of electrolyte 3, which is a conventional one, was measured to be 11.9 mS cm^−1^. Although the ionic conductivity of electrolyte 1, containing anion receptor, is insufficient for commercial use, we found out that the receptor could improve the ionic conductivity in LiCl/organic solvent electrolyte. In dilute solutions, the ionic conductivity of the electrolyte is the sum of the conductivities of the individual ion species as expressed by Equation (3) [22]. Each conductivity is calculated using the product of the three factors below. The factors are the number of carriers contained per unit volume (N), the absolute value of the charge of the ion (Q), and the carrier mobility (u). Conductivity is proportional to each of these three factors. Since Q is constant, it is assumed that N or u, or both, contribute to the increase in ionic conductivity, where u is expressed by the Stokes–Einstein equation (Equation (4)) [23]. It is assumed that the complex anion’s solvation ionic radius (a) was larger than that of Cl^−^ and that of the viscosity (η) of a solution quantitatively increases with the dissolution of the receptor in the organic solvent. Since both the solvated ionic radius (a) and the viscosity (η) are in the denominator of Equation (4), they have negative correlations with u. From the viewpoint of u, it is inferred that the addition of the receptor decreases the ionic conductivity. Despite the above estimation, the ionic conductivity increased with the addition of an anion receptor. This could be attributed to the increase in N, which is the rest of the variables in Equation (3). Therefore, the increase in the ionic conductivity is attributed to the respective ionic conductivities of Li^+^ and Cl^−^. It is suggested that the increase in ion concentration increased the ionic conductivity. To verify whether the concentration of LiCl increased, we measured the Li concentration with ICP-OES.

(3) $$K=\sum_{i}N_{i}Q_{i}u_{i}$$

K: conductivity of material (S cm^−1^).

$N_{i}$: number of carriers (electricity carriers) included in unit volume (cm^−3^).

$Q_{i}$: absolute value of the charge of ion (C).

$u_{i}$: carrier mobility (cm s^−1^/(V cm^−1^)).

(4) $$u=\frac{Ze}{6\pi \eta a}$$

u: mobility (cm s^−1^/(V cm^−1^)), Z: ion charge (-), e: elementary charge (C).

η: viscosity of solution (Pa s), a: radius of solvated ion (cm).

### 3.2. Quantification of Li Concentration in the Electrolyte by Elemental Analysis (ICP-OES)

As shown in Table 3, the Li concentration ($C_{Li}$) of electrolyte 1 (with receptor) was measured to be 1100 ppm, while that of electrolyte 2 (without receptor) was 260 ppm. Converting ppm into M, the Li concentrations (${C^{\prime }}_{Li}$) of electrolyte 1 and electrolyte 2 were 0.17 M and 0.04 M, respectively. The addition of only the 0.05 M receptor could increase the Li concentration by a factor of 4.2. The receptor may be constantly exchanging solvent molecules with several Cl^−^ instead of being stable as an equimolar complex (anion receptor: Cl^−^ = 1:1 (mol/mol)). The ionic bonds between Li cations and anions were weakened by the association of the receptor with Cl^−^. In this case, the mobility of Cl^−^ is expected to be lower than that of Li cation, because the solvated radius of Cl^−^ ($a_{Cl}$) would be larger than that of the Li cation. Li cation can exist more freely. The improvement of the ionic conductivity with the anion receptor is expected to improve the battery performance even with the LiCl electrolyte. 

### 3.3. Initial Charge/Discharge Battery Property and Rate Capability

**Figure 2:**
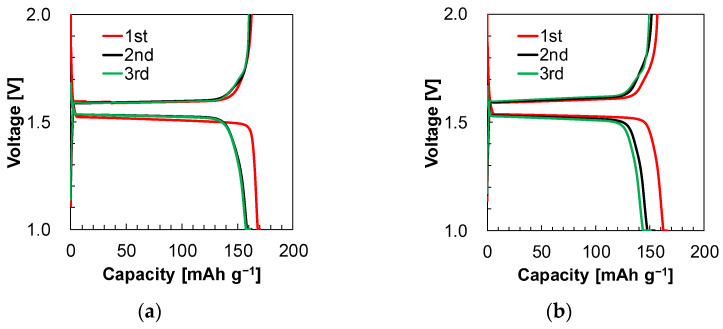
(**a**) Charge/discharge curves for the initial three cycles in the presence of the receptor (electrolyte 1) and (**b**) absence of the receptor (electrolyte 2).

The charge/discharge curves and coulombic efficiencies for the initial three cycles are shown in Figure 2, and the results are summarized in Table 4. A program in which the voltage descends to 1.0 V was defined as charging, and a program in which the voltage rises to 2.0 V was defined as discharging. During charging to 1.0 V, Li^+^ is continuously inserted into the LTO, and Li^+^ is dissolved into the electrolyte from the Li metal surface. During discharging to 2.0 V, Li^+^ de-intercalated from the LTO into the electrolyte, and Li^+^ was deposited on the Li metal surface. 

The first coulombic efficiencies of electrolyte 1 and electrolyte 2 were 95.6% and 94.5%, respectively. The battery with the receptor showed 1.1% higher first coulombic efficiency. The third coulombic efficiencies were 99.2% and 99.1% for electrolyte 1 and electrolyte 2, respectively. In the first cycle, the receptor might have restricted the consumption of LiCl. When we compared both of the first charge curves, the charge curve for electrolyte 1 dropped more sharply to 1.0 V than that for electrolyte 2. 

The difference in the curve shape was due to the fact that electrolyte 1 with the receptor had 3.3 times the ionic conductivity of electrolyte 2. Both batteries had similar charge/discharge curves after the first charge. The decomposition layer of the receptor or solvents increased the resistance of the batteries, and the difference in the cell resistance could be minimized. Another suggested reason is that the concentration polarization is more relieved in electrolyte 1 than in electrolyte 2, perhaps because of the higher Li concentration in electrolyte 1. As a reference, the charge/discharge curves, coulombic efficiencies, and capacities are shown in Figure S4 and Table S1. The shape of the curves was almost the same.

**Figure 3:**
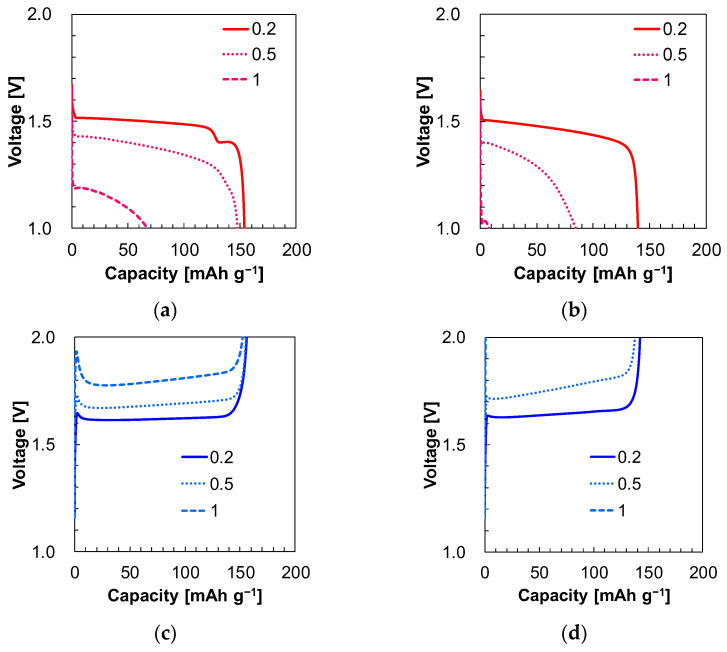
(**a**) Charge rate test curves in the presence of the receptor (electrolyte 1) and (**b**) absence of the receptor (electrolyte 2). (**c**) Discharge rate test curves in the presence of the receptor (electrolyte 1) and (**d**) absence of the receptor (electrolyte 2). The numbers in the figure represent the C rate.

The charge/discharge curves and the capacities in the rate test are shown in Figure 3 and Table 5. As a reference, the rate test curve and capacities are shown in Figure S5 and Table S2. Derivative curves of the rate test curves are shown in Figure S6a–f to clarify the plateau voltage. The charging characteristics were compared first. The battery with electrolyte 1 containing the receptor exhibited two plateaus in the charge curve at 0.2 C (Figure 3a). The first plateau was observed at approximately 1.5 V, while the second one appeared at 1.4 V. These two-step plateaus were not observed for electrolyte 2 without the receptor (Figure 3b). The second plateau at about 1.4 V was flat, indicating a two-phase coexistence reaction similar to LTO [24]. Compared to electrolyte 1 and electrolyte 3, it was observed that the charge curve of electrolyte 1 showed plateaus at 1.4 V and 1.5 V at 0.2 C (Figure S6a). On the other hand, electrolyte 3 only exhibited a plateau at 1.55 V at 0.2 C (Figure S6c). The two-step plateaus only appeared after the battery was kept at a high state of charge. Due to its poor discharge rate property, the battery could hardly discharge at the high rates of discharges. The equilibrium reaction energy could naturally be lower due to the second plateau being located around 0.1 V lower than the first plateau, which relates to the thermodynamic aspect. In terms of the kinetic aspect, the first plateau (≈1.5 V) may have a constant overpotential of 0.1 V. It is believed that the overpotentials are caused by a high resistance layer that may form on the surface of LTO particles. For instance, during the late stage of the charging, the receptors could co-insert with Li^+^. Previous studies have reported that urea can co-intercalate into kaolinite (Al_4_Si_4_O_10_(OH)_8_), which is one of the metal oxide compounds [25,26] such as LTO. The receptor utilized in this study also contains the urea groups. The battery with electrolyte 1 (Figure 3a) showed a charge capacity of about 150 mAh g^−1^ at a rate of 0.5 C. However, the charge capacity dropped to around 60 mAh g^−1^ at 1 C, and even smaller capacities were observed at 2, 3, and 4 C. In contrast, the battery with electrolyte 2 (Figure 3b) showed around 150 mAh g^−1^ charge capacity at 0.2 C. The capacity decreased to about 70 mAh g^−1^ at 0.5 C, and negligible capacities were observed at 1, 2, 3, and 4 C. Therefore, the addition of the receptor into electrolytes improved the charge characteristics. This could have been due to the higher Li concentration in electrolyte 1 compared to electrolyte 2. Even at 4 C, the discharge capacity was 160 mAh g^−1^, corresponding to the high ionic conductivity of the conventional electrolyte (electrolyte 3).

Next, we compared the discharge characteristics. Figure 3c shows the discharge curves of the battery with electrolyte 1. The discharge capacity was around 150 mAh g^−1^ up to 1 C. Almost no capacity was observed at 2, 3, and 4 C. The discharge curves for the battery with electrolyte 2 without the receptor are shown in Figure 3d. The discharge capacity was approximately 140 mAh g^−1^ at 0.2 and 0.5 C. However, at the rate of 1 C, the discharge capacity dropped to 0, as it did at 2, 3, and 4 C. This sudden decline in discharge occurred because the voltage reached the upper cut-off voltage at the beginning of the discharge. During the discharge, the Li^+^ desorption from LTO and Li deposition onto Li metal occurred, resulting in a higher peak voltage at the beginning of the discharge with higher discharge rates. The rate-determining step could be the re-solvation of Li^+^ or desolvation of Li^+^ processes. The receptor contains a glycol unit that might weakly associate with Li^+^. Therefore, the receptor could cause an increase in the resistance of Li^+^ desolvation. 

### 3.4. Cyclability

**Figure 4:**
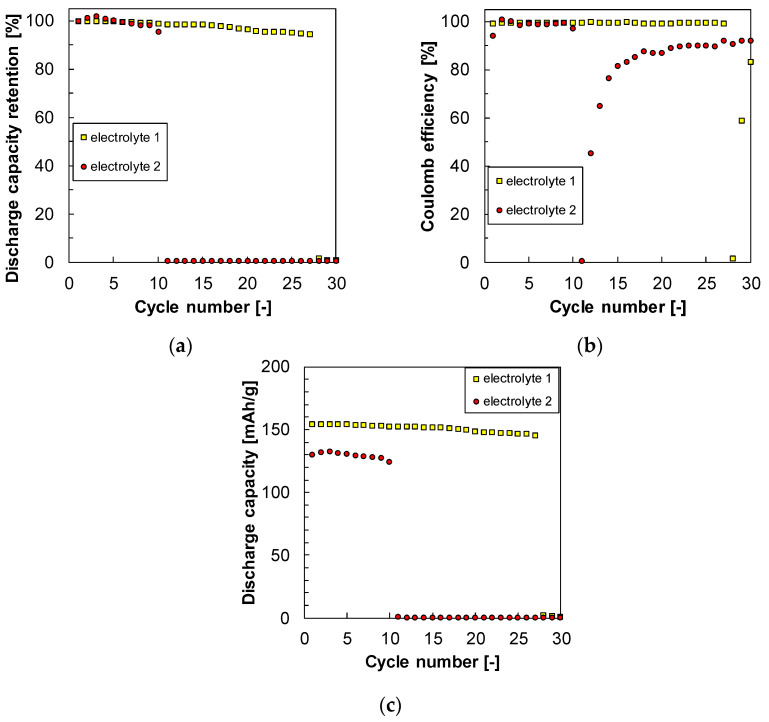

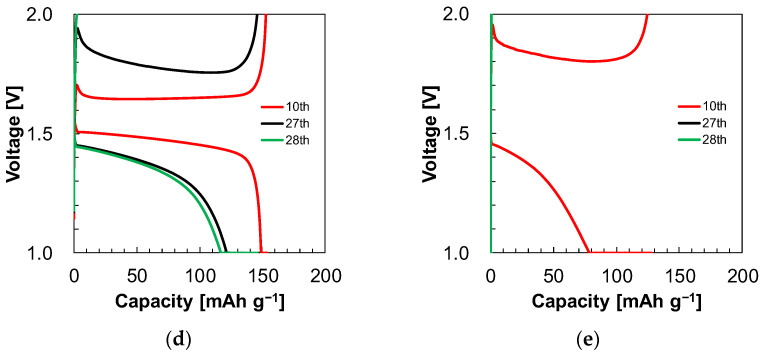
(**a**) Discharge capacity retention rate, (**b**) coulombic efficiency, and (**c**) discharge capacity on the cycle test in the presence (electrolyte 1) and absence (electrolyte 2) of the receptor. (**d**,**e**) The charge/discharge curves in each cycle with electrolyte 1 and electrolyte 2.

Figure 4a demonstrates the discharge capacity retentions for electrolytes 1 and 2 during the 30 cycles. The battery with electrolyte 1 containing the receptor retained a discharge capacity of over 99% until the 27th cycle. However, in the 28th cycle, the discharge capacity dropped sharply to 0%. In contrast, the capacity of the battery with electrolyte 2 deteriorated to around 0% in only the 11th cycle. These results clearly show that the addition of the receptor effectively improved the cyclability. 

The coulombic efficiencies of each battery are shown in Figure 4b. Prior to deterioration, all electrolytes had coulombic efficiencies exceeding 99%. Although the coulombic efficiency for electrolyte 2 appeared to recover after the capacity drop, it only corresponded to the cycles with relatively low capacities. Figure 4c shows the actual discharge capacities during the cycles. The capacity of the battery with electrolyte 2 dropped sharply, reaching almost 0 mAh g^−1^.

We compared the charge/discharge curves before the sudden drop to clarify the reason. Figure 4d shows that the 10th cycle curve had plateaus for the battery with electrolyte 1. However, the 27th cycle, before the deterioration, had a steep charge curve. Additionally, during the 27th discharge, the voltage rose to 1.9 V, close to the cut-off voltage of 2.0 V. The 28th charge curve was similar to the 27th one. However, the discharge curve of the 28th cycle showed no capacity. The voltage quickly rose to the cut-off voltage of 2.0 V, and the discharge was complete. The deterioration of the battery with electrolyte 1 was due to the initial sharp increase in voltage. Similarly, the battery with electrolyte 2 exhibited a sharp voltage increase that occurred in the 11th cycle, as shown in Figure 4e. The presence of the receptor reduced the rapid voltage rise resulting from the initial resistance of the battery.

During repeated charge/discharge cycles, a gradient in the concertation of lithium ions may form in the LTO electrode. In this experiment, the rest time between each charge and discharge was 15 min, which was the same as the first charge and discharge. No slopy curves or large initial resistance in the discharge curves were observed within the first three cycles. The rest time was sufficient to allow the concentration gradient in the electrode and electrolyte to relax. The slopy curves and the large initial resistance are not caused by reversible kinetic factors such as the Li^+^ concentration gradient. Instead, they may be caused by irreversible factors that depend on the preceding cycles. After the cycle test, the Nyquist-plot semi-circles were observed (Figure S7), indicating an irreversible change. If LiCl is continuously consumed, the salt concentration will decrease as the cycles proceed. For LiCl instead of LiPF_6_, it is well known that the Cl^−^ is reactive by oxidation to form Cl_2_. The potential range on the LTO electrode is 2.0 V–1.0 V (vs. Li/Li^+^). Li standard electrode potential is −3.04 V (vs. SHE) (Li^+^ + e^−^ = Li), and the Cl standard electrode potential is 1.358 V (vs. SHE) (Cl_2_ (g) + 2e^−^ = 2Cl^−^) [27]. The electrode potential of Cl^−^ is 4.398 V (vs. Li/Li^+^). Based on the experiment’s potential range (1.0 V–2.0 V), it is unlikely that oxidative decomposition occurred. 

EC could be reductively decomposed on the Li metal electrode, and LiCl could also be combined into the solid electrolyte layer (SEI). LiCl may be continuously consumed during the consumption of EC on the Li metal side in the reductive environment. Although LiPF_6_ is usually present in excess amounts in electrolytes such as 1.0 M LiPF_6_, the effect of salt consumption on ionic conductivity is limited in the short term. However, a decrease in LiCl might disturb the battery performance due to the low concentrations in electrolyte 1 (0.17 M) and electrolyte 2 (0.04 M). In this sense, Li is relatively concentrated.

The extended cycle characteristics in electrolyte 1 are attributed to be (1) the Li salt concentration having increased due to the addition of the receptor (quantitative aspect) and (2) the receptor associating with Cl^−^ to form a complex with a large solvation radius, which can kinetically disturb the decomposition on the reduction side (qualitative aspect). Besides LiCl consumption, one of the candidates for battery deterioration could be Al corrosion. It has been reported that Al corrosion occurs around 3.5 V (Li/Li^+^) with LiTFSI, which forms no passivation layer like AlF_3_ [28]. In general, with the electrolyte containing sufficient LiPF_6_, the Al corrosion does not occur up to 5.0 V (Li/Li^+^) due to the passivation layer, AlF_3_ [29]. Here, we set 1.0 V to 2.0 V as the voltage range. It is suggested that the Al corrosion is unlikely to occur even without LiPF_6_. As for the receptor-containing electrolytes, there is also another possibility that the receptors with urea moieties act as a kind of antirust agent. It should be mentioned that the suppression of Al corrosion by urea has been calculated [30]. 

Although we used the LTO electrode in this report, in the future, the electrolyte containing LiCl and an anion receptor could be applied to the batteries with a higher voltage. Indeed, a lower oxidative decomposition potential was observed for electrolyte 1 (4.0 V) than electrolyte 2 (4.5 V) during the linear sweep voltammetry (LSV) experiment (SUS|electrolyte with poly propylene separator|Li) (Figure S8). The receptor increased the oxidative stability of the LiCl-type electrolyte. It is suggested that the receptor contributed to the higher oxidative stability of the Cl^−^ due to the association. We will conduct further experiments to elucidate the function of the receptor in electrolytes. Understanding the receptor’s working mechanism will help us design the anion receptor for better application.

## 4. Conclusions

In this study, we elucidated that the addition of the anion receptor into the saturated LiCl EC/DMC improved the properties of the electrolyte and the battery. The ionic conductivity of the saturated LiCl EC/DMC increased 3.3 times from 0.023 to 0.075 mS cm^−1^. This enhancement can be explained by the fact that the saturated LiCl concentration increased 4.2 times from 0.04 M to 0.16 M (ICP-OES) in the presence of the receptor. The addition of the anion receptor also improved the rate capability and the cyclability. In terms of charge rate capability, the battery with the anion receptor showed a capacity of around 148 mAh g^−1^ at the rate of 0.5 C, but the battery without the receptor showed only 85 mAh g^−1^. For the discharge rate capability, the battery with the receptor showed a discharge capacity of 152 mAh g^−1^ at the rate of 1 C, while the battery without the receptor showed 0 mAh g^−1^. In the cycle test, the battery with the receptor could be cycled up to the 27th cycle at around 150 mAh g^−1^. The discharge capacity of the battery without the receptor dropped to 0 mAh g^−1^ in the 11th cycle. From the perspective of structural improvement of the receptor, the future plan includes two crucial aspects: enhancing the ability to associate with anions and improving the solubility in organic solvents that constitute the electrolyte. By systematically examining various structures, we aim to explore the relationship between these factors and the potential to achieve electrolyte performance comparable to commercial electrolytes. Further optimization of the anion receptor is expected to lead to the commercial use of inexpensive and environmentally friendly LiCl electrolytes. In this report, we primarily approached the proof-of-concept by examining how the addition of anion receptors improves the characteristics of the electrolyte. In addition to examining the electrolyte properties, we will also study the properties of electrodes using scanning electron microscopy (SEM) and X-ray photoelectron spectroscopy (XPS) in due course.

## Figures and Tables

**Table 1:** The prepared electrolytes.

Item	Salt	Solvent	Anion Receptor	State
Electrolyte 1	LiCl	EC/DMC = 1/1 (*v*/*v*)	0.05 M	LiCl saturated
Electrolyte 2			none	
Electrolyte 3	LiPF_6_		none	1 M

**Table 2:** The ionic conductivity of electrolytes.

Electrolytes	Ionic Conductivity (mS cm^−1^)	Ratio of Ionic Conductivity
Electrolyte 1	0.075	3.3
Electrolyte 2	0.023	1
Electrolyte 3	11.9	517

**Table 3:** The Li concentrations measured by ICP-OES.

Electrolytes	$\mathrm{Li}\;\mathrm{Concentration}\;(C_{Li}$) (ppm)	$\mathrm{Li}\;\mathrm{Concentration}\;({C^{\prime }}_{Li})$ (M)	Ratio of Li Concentration
Electrolyte 1	1100	0.17	4.2
Electrolyte 2	260	0.04	1

**Table 4:** The initial charge/discharge battery properties.

Electrolytes	Coulombic Efficiency (%)			Charge Capacity (mAh g^−1^)			Discharge Capacity (mAh g^−1^)		
	First	Second	Third	First	Second	Third	First	Second	Third
Electrolyte 1	95.6	99.2	99.2	170.2	162.4	161.4	162.6	161.1	160.0
Electrolyte 2	94.5	98.1	99.1	166.1	154.4	150.6	156.9	151.5	149.2

**Table 5:** The charge capacities and discharge capacities at each rate of 0.2, 0.5, and 1 C ^1^.

Electrolytes	Charge Capacity (mAh g^−1^)			Discharge Capacity (mAh g^−1^)		
	0.2 C	0.5 C	1 C	0.2 C	0.5 C	1 C
Electrolyte 1	154	148	67	156	155	152
Electrolyte 2	140	85	8	142	138	0

^1^ At the rates of 2, 3, and 4 C, all the capacity was 0 mAh g^−1^.

## Data Availability

The original contributions presented in the study are included in the article, further inquiries can be directed to the corresponding author.

## Supplementary Materials

The following supporting information can be downloaded at https://www.mdpi.com/article/10.3390/ma17133244/s1. Figure S1: The structure of a coin cell. Figure S2: (a) Front view of the four-electrode cell for the ionic conductivity measurement, and (b) cross-section of the cell (in detail: https://ec-frontier.co.jp/product/cell/SB1400.php (accessed on 15 June 2024)). Figure S3: Nyquist plots for electrolyte 1 (with receptor), electrolyte 2 (without receptor), and electrolyte 3 (conventional). Figure S4. Charge/discharge curves for the initial three cycles in the conventional electrolyte (electrolyte 3). Figure S5: (a) Charge rate test curves in the presence of the receptor (electrolyte 3) and (b) discharge rate test curves in electrolyte 3 (the numbers in the figure represent the C rate). Figure S6. (a) Derivative curves of the charge rate test curves in the presence of the receptor (electrolyte 1), (b) absence of the receptor (electrolyte 2), (c) and conventional electrolyte (electrolyte 3). (d) Derivative curves of discharge rate test curves in the presence of the receptor (electrolyte 1), (e) absence of the receptor (electrolyte 2), and (f) conventional electrolyte (electrolyte 3). The numbers in the figure represent the C rate. (The derivative curves were normalized with each max value; for some 1 C rate curves, derivative curves could not be obtained well because almost no capacities were obtained.). Figure S7. (a) Nyquist plots after initial three cycles for electrolyte 1 (with receptor) and electrolyte 2 (without receptor), and (b) Nyquist plots after cycle test. Figure S8. (a) Linear sweep voltammetry curves for electrolyte 1 (with receptor) and electrolyte 2 (without receptor) at a scan rate of 0.1 mV s^−1^ at 25 °C from OCV to 6.0 V, and (b) from OCV to −0.5 V (the cells were the 2 electrode-type cells (SUS|electrolyte with poly propylene separator|Li)). Table S1. The initial charge/discharge battery properties for the conventional electrolyte (electrolyte 3). Table S2. The charge capacities and discharge capacities at each rate of 2, 3, and 4 C.
