# Supplementary material for: Unlocking the Use of LiCl as an Inexpensive Salt for Lithium-Ion Batteries with a Novel Anion Receptor
Source: Materials (Basel). 2024 Jul 2;17(13):3244. doi: 10.3390/ma17133244 (PMC11242702; doi:10.3390/ma17133244)
Supplement: Supplementary file 1 [file materials-17-03244-s001.zip › materials-3031379-supplementary.pdf]

Supporting Information

Unlocking the Use of LiCl as an Inexpensive Salt for Lithium-Ion Batteries with a Novel Anion Receptor

Manabu Hirasawa [1,2,*], Akihiro Orita [1], Tsubasa Mimuro [2] and Shin-ichi Kondo [2,*]

[1] Institute for Advanced Integrated Technology, Resonac Corporation, 48 Wadai, Tsukuba 300-4247, Ibaraki, Japan; orita.akihiro.xiasy@resonac.com
[2] Department of Chemistry, Faculty of Science, Yamagata University, Yamagata 990-8560, Yamagata, Japan;
s221702d@st.yamagata-u.ac.jp
* Correspondence: hirasawa.manabu.xicfq@resonac.com or s2131272d@st.yamagata-u.ac.jp (M.H.); kondo@sci.kj.yamagata-u.ac.jp (S.-i.K.)

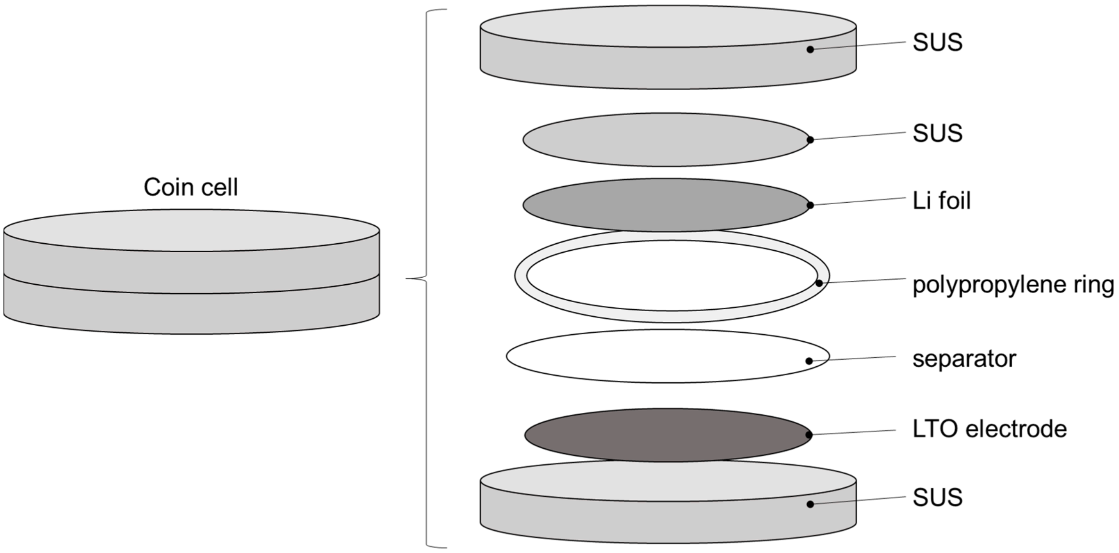

Figure S1. The structure of a coin cell.

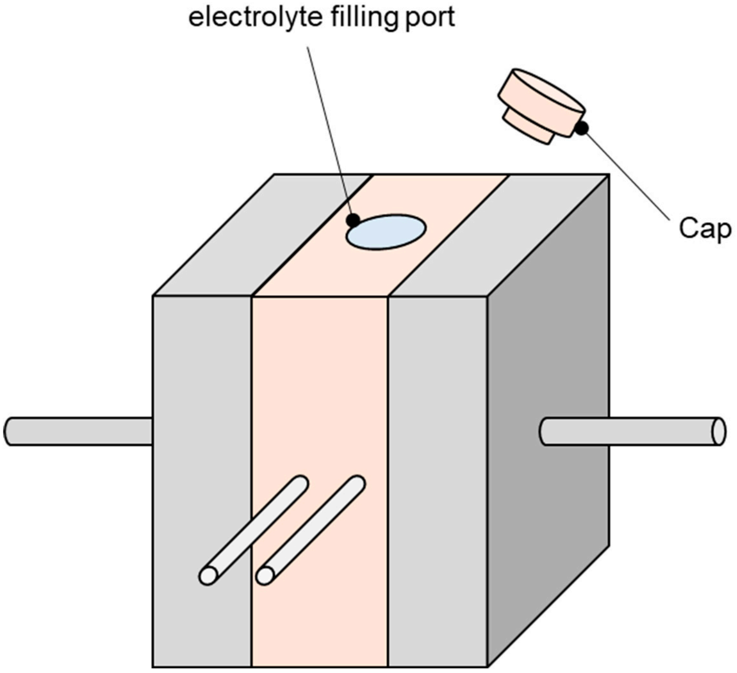

(**a**)

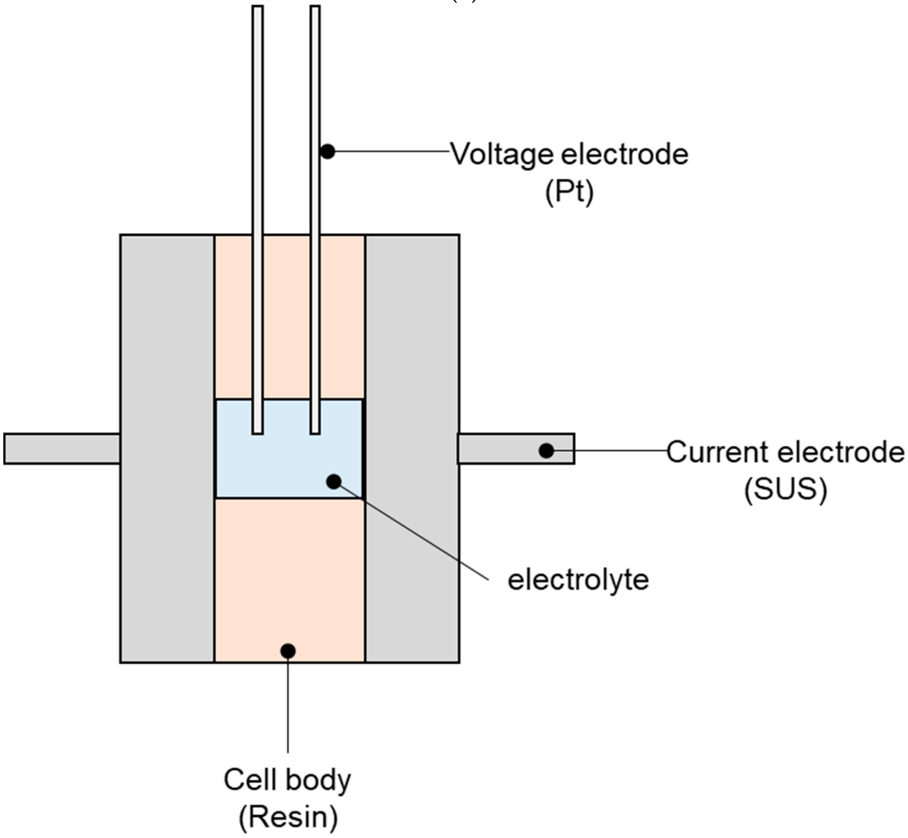

(b)

Figure S2. (a) Front view of the four-electrode cell for the ionic conductivity measurement, and (b) cross section of the cell (in detail: https://ec-frontier.co.jp/product/cell/SB1400.php (accessed on 15 June 2024)

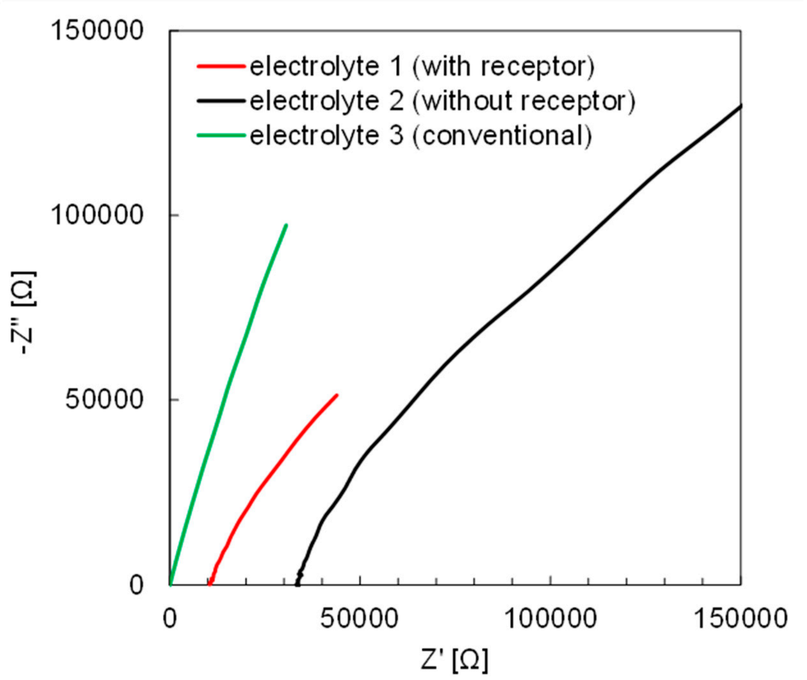

Figure S3. Nyquist plots for electrolyte 1 (with receptor), electrolyte 2 (without receptor), and electrolyte 3 (conventional).

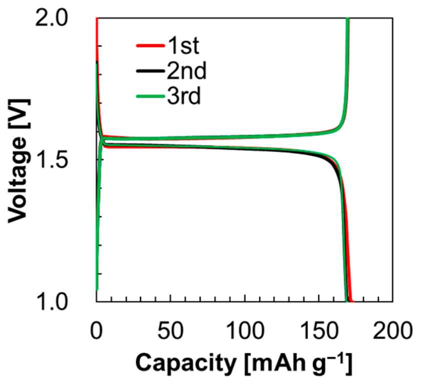

Figure S4. Charge/discharge curves for the initial three cycles in the conventional electrolyte (electrolyte 3).

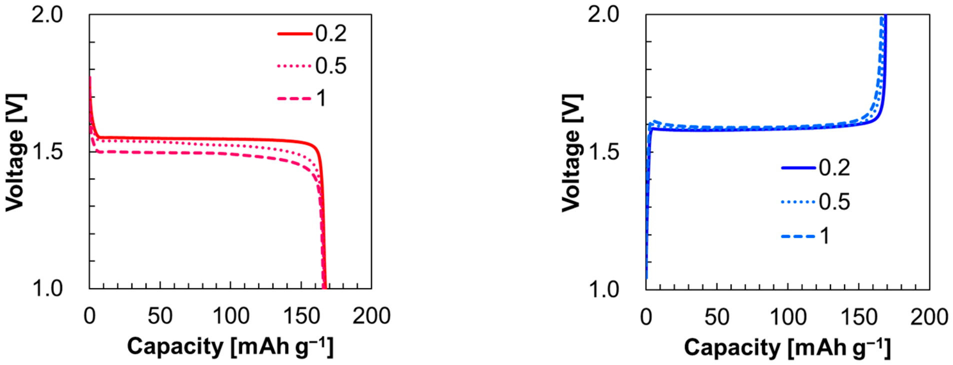

(a) **(b)**

Figure S5. (a) Charge rate test curves in conventional electrolyte (electrolyte 3), and (b) discharge rate test curves in conventional electrolyte (electrolyte 3) (the numbers in the figure represent the C rate.).

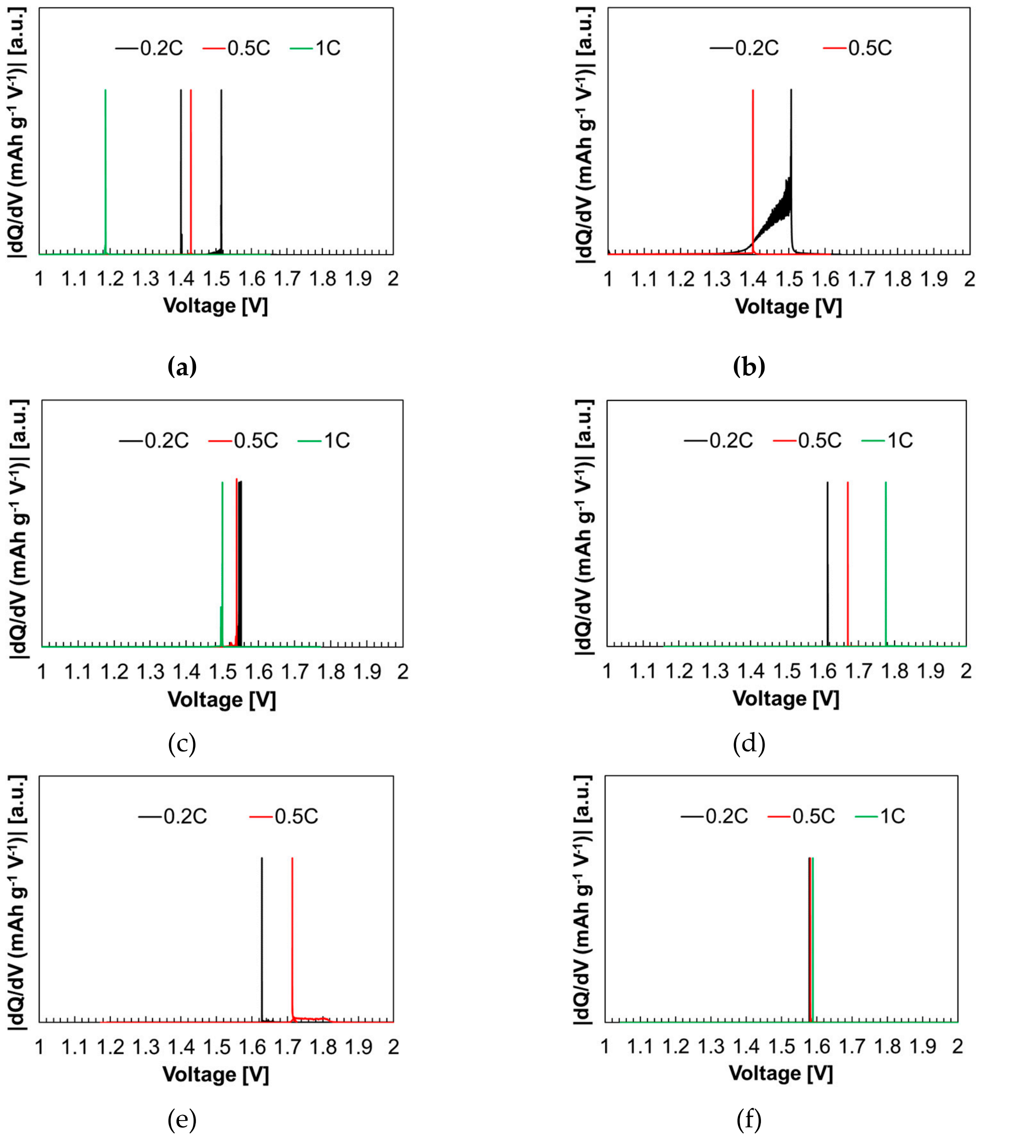

Figure S6. (a) Derivative curves of the charge rate test curves in the presence of the receptor (electrolyte 1), (b) absence of the receptor (electrolyte 2), (c) and conventional electrolyte (electrolyte 3). (d) Derivative curves of discharge rate test curves in the presence of the receptor (electrolyte 1), (e) absence of the receptor (electrolyte 2), and (f) conventional electrolyte (electrolyte 3). The numbers in the figure represent the C rate. (The derivative curves were normalized with each max value; for some 1C rate curves, derivative curves could not be obtained because almost no capacities were obtained.).

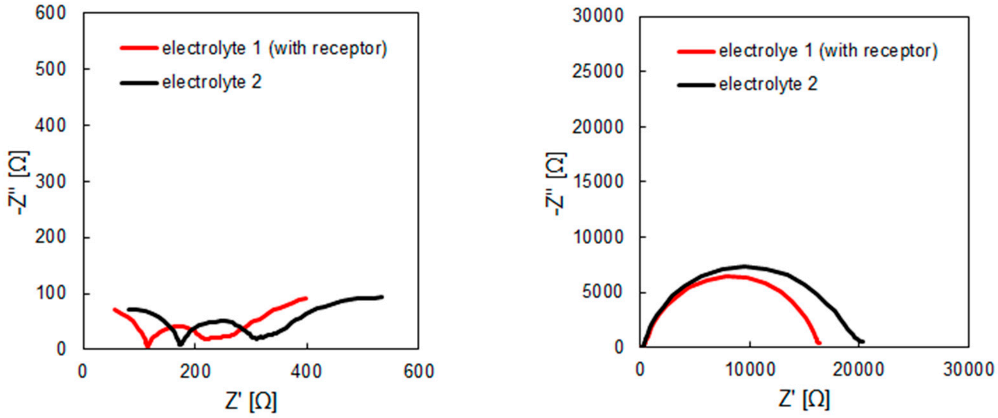

(a) **(b)**

Figure S7. (a) Nyquist plots after initial three cycles for electrolyte 1 (with receptor) and electrolyte 2 (without receptor), and (b) Nyquist plots after cycle test.

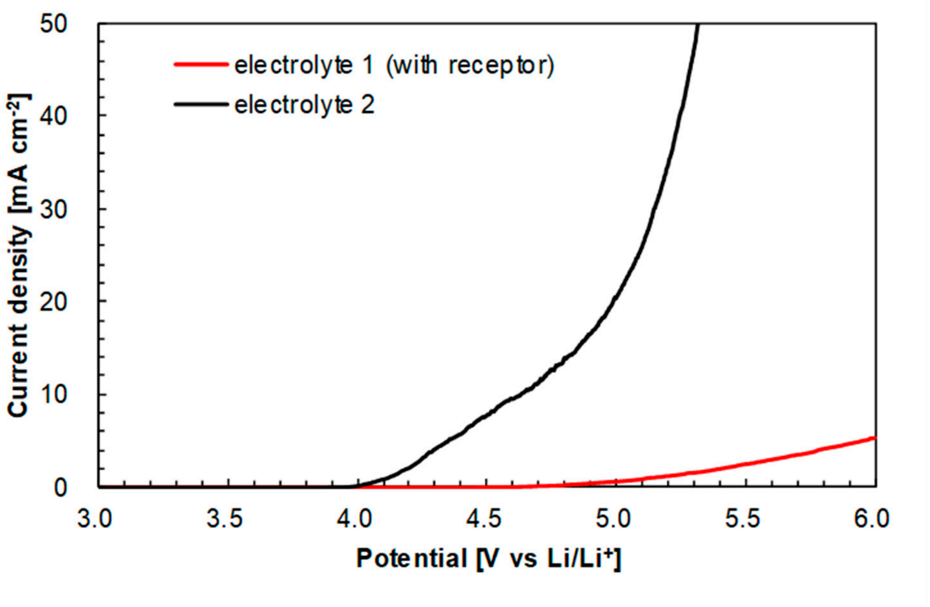

(**a**)

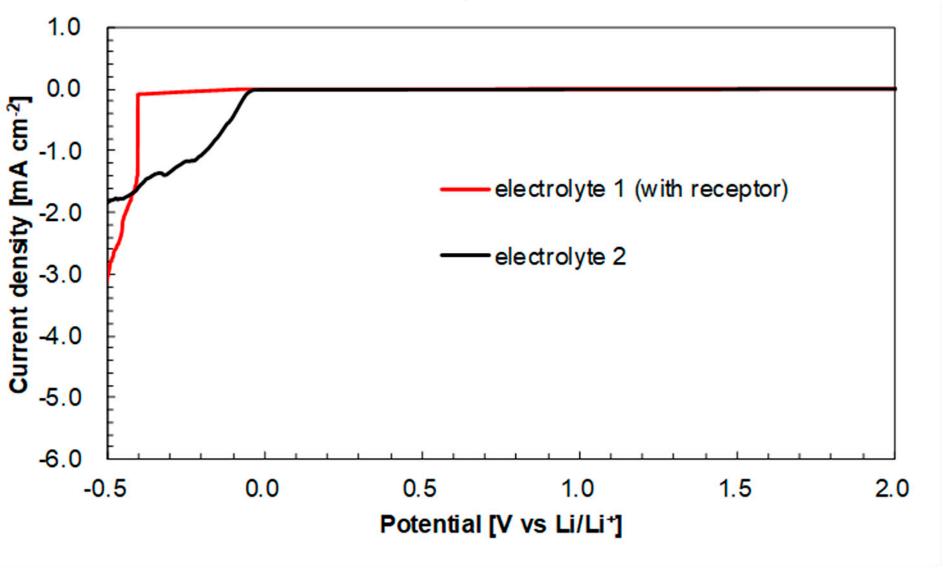

(**b**)

Figure S8. (a) Linear sweep voltammetry curves for electrolyte 1 (with receptor) and electrolyte 2 (without receptor) at a scan rate of 0.1 mV s^{-1} at 25 °C from OCV to 6.0 V, and (b) from OCV to −0.5 V (the cells were the 2 electrodes-type cells (SUS | electrolyte with poly propylene separator | Li)).

Table S1. The initial charge/discharge battery properties for the conventional electrolyte (electrolyte 3).

**Electrolytes**	**Coulombic efficiency (%)**			**Charge capacity (mAh g^{-1})**			**Discharge capacity (mAh g^{-1})**		
	**1st**	**2nd**	**3rd**	**1st**	**2nd**	**3rd**	**1st**	**2nd**	**3rd**
Electrolyte 3	98.1	99.6	99.8	173.0	170.1	169.7	169.7	169.4	169.3

Table S2. The charge capacities and discharge capacities at each rate of 2, 3, and 4 C.

**Electrolytes**	**Charge capacity (mAh g^{-1})**			**Discharge capacity (mAh g^{-1})**		
	**2 C**	**3 C**	**4 C**	**2 C**	**3 C**	**4 C**
Electrolyte 1	0	0	0	0	0	0
Electrolyte 2	0	0	0	0	0	0
Electrolyte 3	164.6	163.5	162.3	164.5	161.8	160.2
